# Acid Sphingomyelinase Deficiency: A Clinical and Immunological Perspective

**DOI:** 10.3390/ijms222312870

**Published:** 2021-11-28

**Authors:** Carolina Pinto, Diana Sousa, Vladimir Ghilas, Andrea Dardis, Maurizio Scarpa, Maria Fatima Macedo

**Affiliations:** 1 Addiction Biology, Instituto de Investigação e Inovação em Saúde (i3S), Faculdade de Medicina, Universidade do Porto, 4050-091 Porto, Portugal; cpinto@i3s.up.pt; 2 Department of Medical Sciences, University of Aveiro, 3810-193 Aveiro, Portugal; dianacgs@ua.pt (D.S.); ghilasvladimir@ua.pt (V.G.); 3 Regional Coordinating Center for Rare Diseases and MetabERN University Hospital, 33100 Udine, Italy; andrea.dardis@asufc.sanita.fvg.it (A.D.); maurizio.scarpa@metab.ern-net.eu (M.S.); 4 Cell Activation and Gene Expression Group, Instituto de Biologia Molecular e Celular (IBMC), Instituto de Investigação e Inovação em Saúde (i3S), Universidade do Porto, Rua Alfredo Allen, 208, 4200-135 Porto, Portugal

**Keywords:** Niemann–Pick, acid sphingomyelinase deficiency, sphingomyelinase, lysosomal storage disease, immune

## Abstract

Acid sphingomyelinase deficiency (ASMD) is a lysosomal storage disease caused by deficient activity of acid sphingomyelinase (ASM) enzyme, leading to the accumulation of varying degrees of sphingomyelin. Lipid storage leads to foam cell infiltration in tissues, and clinical features including hepatosplenomegaly, pulmonary insufficiency and in some cases central nervous system involvement. ASM enzyme replacement therapy is currently in clinical trial being the first treatment addressing the underlying pathology of the disease. Therefore, presently, it is critical to better comprehend ASMD to improve its diagnose and monitoring. Lung disease, including recurrent pulmonary infections, are common in ASMD patients. Along with lung disease, several immune system alterations have been described both in patients and in ASMD animal models, thus highlighting the role of ASM enzyme in the immune system. In this review, we summarized the pivotal roles of ASM in several immune system cells namely on macrophages, Natural Killer (NK) cells, NKT cells, B cells and T cells. In addition, an overview of diagnose, monitoring and treatment of ASMD is provided highlighting the new enzyme replacement therapy available.

## 1. Introduction

Acid sphingomyelinase deficiency (ASMD) is a lysosomal storage disease (LSD) caused by deficient activity of the acid sphingomyelinase (ASM) enzyme, leading to the accumulation of sphingomyelin. This disease is characterized by foam cell infiltration in different tissues, lipid storage and clinical manifestations which may overlap: pulmonary insufficiency, hepatosplenomegaly and neurodegeneration [1]. These broad clinical manifestations make it hard to clinically distinguish ASMD from other LSDs, such as Gaucher disease.

The first sighting of ASMD was reported by Albert Niemann, in 1914, in an infant patient, but it was not until 1927 that the disease was considered clinically differentiated from Gaucher disease by Ludwig Pick, who reviewed reports of infants with rapidly progressive neurodegenerative disorders [1]. In subsequent years, the disease became known as Niemann–Pick disease (NPD). In 1934, the lipid accumulated in this disease was identified as sphingomyelin, but only in 1966, the characterization of the deficiency in human-sphingomyelin-cleaving enzyme was experimentally performed in NPD-patient samples [2]. Subsequent clinical and biochemical studies identify a singular group of NPD patients that were designated as NPC. In 1985, it was proven, through cell culture from NPC patients cells, that the metabolic defect in NPC is clearly distinguishable from sphingomyelin cleaving enzyme defect, identifying a cholesterol trafficking defect [3]. Nowadays, we know that NPC is caused by defects in one of two proteins involved in the lysosomal transport of unesterified cholesterol (NPC1 and NPC2).

Today, we know that NPDs are divided into two separate groups: acid sphingomyelinase deficiency (ASMD)—comprising the ASMD A and B—and Niemann–Pick type C (NPC) [4].

ASMD results from mutations on the *SMPD1* gene (MIM# 607608), encoding for ASM. The deficiency of this enzyme leads to accumulation of lipids, mainly sphingomyelin, in various tissues throughout the body, and this is responsible for the observed phenotypes. ASMD A and B presentations are different, so the fate of the patients is affected. ASMD A is considered to be the direst form of ASMD, as it is fatal. It is characterized by early infancy onset and little to no ASM residual activity, which leads to rapidly progressive systemic manifestation symptoms—mainly hepatosplenomegaly—and severe central nervous system impairment. Death is unavoidable and typically occurs within the first 3 years of age [5]. On the other hand, ASMD B is characterized by an irregular age onset, a slow progressive systemic apparition of symptoms and, in general, no neurological involvement. Hepatosplenomegaly is commonly the first clinical presentation, but decreased pulmonary and liver function over time are also noteworthy [5,6]. Because of the broad spectrum of the disease’s severity, the lifespan of patients is highly variable, ranging from very early death to long burdened lives [7].

Herein we review the current knowledge on this disease, with a focus on the immune-system alterations present and therapeutic approaches unfolding.

## 2. Cellular Alterations

Under normal conditions, the ASM enzyme contributes towards cellular homeostasis by participating in membrane degradation and turnover. This is because sphingomyelin, the substrate of ASM enzyme, is a major membrane component. When degraded, sphingomyelin is turned into phosphocholine and ceramide, a crucial signaling lipid. In patients with ASMD, the low levels of this enzyme lead to sphingomyelin accumulation and consequently to the apparition of lipid laden cells designated as foam cells. These are usually macrophages. However, tissue-specific cells have been reported to be affected as well. Foam cells have been reported to be present in the lungs, spleen (that can reach 10 times in normal size in ASMD patients), bone marrow, lymph nodes and liver [7], and although rare, they can be found in mucosal and submucosal small and large intestines [8]. Lipid laden cells eventually lose all function and die, causing multiple clinical features.

## 3. Clinical Presentation and Diagnosis

ASMD clinical manifestations represent the first line of differential diagnosis of this disease, which presents itself as a multi-organ disease [7]. Hepatosplenomegaly and lung dysfunction are common symptoms in ASMD and are accompanied by central nervous system involvement in the case of ASMD A. ASMD A has a rapid progression and is mostly fatal in the first years of life. ASMD B is characterized by a slow progression, with milder symptoms in which patients often reach adulthood.

Regarding hepatosplenomegaly, it can range from mild to severe, and it is normally associated with hepatic fibrosis and increased levels of transaminases [9]. The characteristic increase of hepatic volume is due to the accumulation of sphingomyelin in Kupffer cells and hepatocytes [10]. Liver disease can lead to fatal cases of liver failure [1]. Splenomegaly can also be present and is the result of infiltration by lipid-laden macrophages [7]. This is normally associated with abdominal pain/discomfort, splenic infarctions and secondary cytopenias [1,7]. The second most common clinical characteristic of ASMD is lung dysfunction, which ranges from none to severe oxygen dependence [1]. Finally, regarding the central nervous system involvement, it is a common feature of ASMD A that leads to a rapid and progressive neurodegenerative course of the disease [7]. Traditionally, ASMD B has been categorized as non-neuropathic. However, several reports have described patients with subtle intermediate forms who satisfy criteria for ASMD B but who also have some neurologic manifestations, namely ataxia, learning difficulties and motor delay [11,12].

ASMD A patients can also present severe hypotonia and a cherry-red spot in the macula [1,7]. This last one can also be present in ASMD B patients, but it is not very common, although a reddish-brown halo surrounding the macula in the eyes may appear [1].

ASMD B patients, in contrast to ASMD A patients, present mixed dyslipidemia, namely increased levels of triglycerides, LDL and VLDL cholesterol, whereas HDL cholesterol levels are decreased [1,7]. Patients with ASMD B often have a growth restriction, which is associated with a delayed bone age, and a delayed onset of puberty [1]. Besides all of these features, ASMD B also presents a higher phenotypic heterogeneity than ASMD A patients [7].

**Figure 1:**
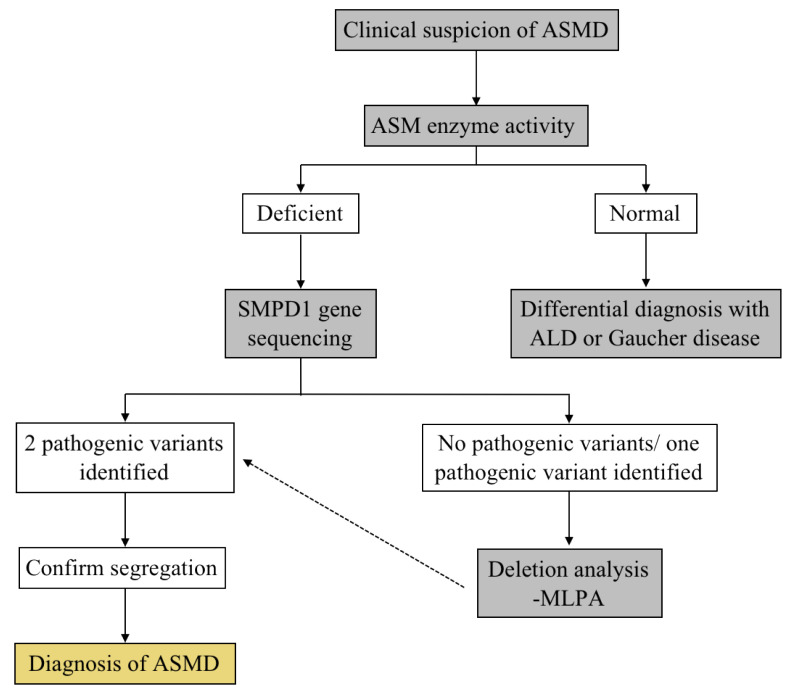
Differential diagnosis for ASMD.

The clinical manifestations of ASMD are the first reason that leads to the suspicion of this disease. However, these clinical features can also be present in other diseases, including primary hepatic disease and other LSD associated with distinct hepatosplenomegaly, such as Gaucher disease, acid lipase deficiency, Niemann–Pick C [9]. With this in mind, the diagnosis of ASMD is based on the demonstration of low or absent ASM enzymatic activity in the patient’s cells (lower than 10–15% of the activity measured in healthy controls) [9]. Enzymatic activity can be measured by using isolated leukocytes, dried blood spots or cultured skin fibroblasts [9]. After the confirmation of the ASMD diagnosis, a genetic analysis SMPD1 gene is performed in order to confirm the diagnosis and provide genetic counseling to the families [10]. Diagnostic algorithms, such as the one presented in Figure 1, help to differentially diagnose this disease.

## 4. Molecular Genetics

ASMD is pan-ethnic and, as such, is spread out to various ethnic groups [9]. However, the ASMD A phenotype presents a higher frequency in Ashkenazi Jewish population [13,14].

The disease is caused by pathogenetic variants in the *SMPD1* gene (MIM# 607608), located in chromosome 11p15.4 [15] and is inherited as a recessive trait.

The gene is 5 kb long and consists in six exons. Two in-frame functional start codons were identified at codons 1 and 33. Site-directed mutagenesis and expression studies demonstrated that both ATGs are functional in vitro [16]. However, several lines of evidence suggest that in vivo translation of wild-type ASM initiates from the first in-frame ATG [17,18,19].

To date, more than 200 variants within the *SMPD1* have been described in patients affected by ASMD (see the Human Gene Mutation Database (http://www.hgmd.org, accessed on 5 October 2021)). Gene alterations include missense, nonsense, small deletions or insertions and splicing abnormalities. However, missense mutations represent the most common type of pathogenic variant found [13]. In addition, several *SMPD1* polymorphisms have been reported [1,20].

The spectrum of *SMPD1* mutant pathogenetic variants has been described in patients from specific populations, such as Italy [21,22], Spain [23], Turkey [16,24], The Czech Republic and Slovakia [12], China [19], The Netherlands and Belgium [25]. The most frequently reported mutation worldwide is a three-base deletion, leading to the loss of the arginine residue Arg610del (p. R610del). However, the reported allele frequencies of this mutation among patients range from 100% in Canary Islands [26] to only 9.4% in Italy [22], where the most frequent mutation was the nonsense variant p.W32X, representing 18.8% of ASMD B alleles [22]. Interestingly, the p.R610del mutation has not been found in patients from China [19] or the Czech Republic [12].

In addition, few mutations are more frequently represented among individuals of a particular ethnic group. For example, three mutations, namely p.F333SfsX52, p.L304P and p.R498L, account for about 90% of mutated alleles in patients affected by ASMD A in the Ashkenazi Jewish population [13].

Both the p.H423Y and p.W393G variants, which are present in 75% and 100% of ASMD B alleles in Saudi Arabians and Romani people, respectively, seem to be associated with the intermediate phenotype [16,27].

Finally, the p.Q294K mutation has been found in patients from different populations. However, it is highly frequent in patients from Czech and Slovak heritage [12].

### Genotype–Phenotype Correlations

The disease severity is often correlated with the type of inherited *SMPD1* variant [13,16]. In general, the presence of a mild mutation leading to the synthesis of a partially active ASM protein in one allele would be enough to prevent the development of the severe ASMD A phenotype [13]. However, the spectrum of *SMPD1* mutations is extremely heterogeneous; most mutations have been found in single families and as compound heterozygous. Therefore, it is quite difficult to correlate the genotype with the phenotype. Nevertheless, some assumptions can be made based on functional analysis of single mutants and for few recurrent mutations found in homozygosity [13].

Indeed, the most frequently reported mutation worldwide is a three-base deletion which leads to the loss of the arginine residue p.Arg610del (p.R610del) and is strongly associated with attenuated ASMD B phenotype. The p.F333SfsX52, p.L304P and p.R498L, highly frequent among Ashkenazi Jewish patients, are associated to the severe ASMD A, while p.H423Y, p.W393G and the p.Q294K variants seem to be associated with the intermediate phenotype [16,27].

In general, mutations that create a premature stop codon, such as nonsense and small deletions or insertions that cause a shift of the open reading frame, would lead to the generation of mRNA species rapidly eliminated by nonsense mediated decay (NMD) or to the synthesis of truncated and likely non-functional proteins. Therefore, although found in few patients, these kinds of variants are considered to be severe [14].

An exception to this general consideration is the nonsense variant p.W32X, associated with the ASMD B phenotype. This variant would lead to the synthesis of a very short and probably instable peptide. To explain the unexpected clinical phenotype observed in patients carrying this variant, it has been hypothesized that, when the first ATG is unable to produce a canonical transcript, the second initiation codon (ATG33) may be used, resulting in the synthesis of a protein missing the first 32 residues of the predicted signal peptide but still partially active. A similar assumption can be proposed for the p.R3AfsX76 variant, quite frequently found among ASMD B Chinese patients, even in homozygous status [19]. This hypothesis has been supported by in vitro expression studies of the c.2T>G mutation, which results in the substitution of the first methionine to threonine. This variant is predicted to inactivate the first in-frame translation start site. However, when expressed in vitro, a high residual activity was detected (26.9% of wild type) [18], confirming that, in vitro, in the absence of the first ATG, the second one (ATG33) can serve as a translation initiation site [28]. In agreement with these data, patients homozygous for this mutation displayed a mild form of the disease [22].

Besides these general correlations, it is worth noting that a phenotypic heterogeneity has been described among patients with the same genotype. This might be explained, at least in part, by epigenetic factors. Indeed, it has been reported that the *SMPD1* locus is paternally imprinted [29], meaning that the gene is normally expressed by the maternal chromosome, as the paternal chromosome is inactivated [29]. Thus, it is possible that two heteroallelic individuals presenting the same genotype may present different phenotypes, depending on whether the severe mutation is expressed in the paternal or the maternal chromosome [29].

## 5. Acid Sphingomyelinase: Structure and Lysosomal versus Cellular Membrane Activity

The *SMPD1* gene gives rise to two ASMs: the lysosomal sphingomyelinase and the secretory sphingomyelinase that is released extracellularly and has increased susceptibility to Zn^2+^ ions [30,31,32]. Originally, it was proposed that the two ASMs were generated via differential trafficking of a common protein precursor. However, it has been recently hypothesized that secretory ASM might be generated by lysosomal exocytosis [33]. Independently of the mechanism leading to ASM translocation to the cellular membrane, it is recognized that internal and external stressors leads to a rapid transport of the enzyme to the cellular membrane [33,34]. Pathogens, radiation, oxidative stress and cytokines are among the stressors triggering extracellular ASM activity [33].

ASM protein consists of a N-terminal saposin domain and a C-terminal catalytic domain. These two domains are linked by a connector region. The catalytic domain has phosphoesterase activity, whereas the build-in saposin domain facilitates lipid isolation for subsequent cleavage by the catalytic domain [35]. The presence of the build-in saposin domain makes ASM an exception in the glycosphingolipid lysosomal hydrolases in its requirement of activating proteins. Indeed, ASM is able to hydrolase sphingomyelin in the absence of exogenous saposins [36]. However, ASM activity can be stimulated by the presence of saposin D [37,38].

In 2016, the crystal structure of mouse and human ASM was obtained by two independent groups [39,40]. The enzyme has the shape of a large shallow bowl. The saposin domain consists of four α-helices stabilized by three disulphide bonds. The connector is a rigid region that is rich in proline residues and links the saposin domain with the catalytic domain. The C-terminal catalytic domain is characterized by two six-stranded mixed β-sheets surrounded by eight α-helices. The catalytic domain possesses an additional C-terminal subdomain consisting of four α-helices. The catalytic domain forms a spherical domain at the base of which resides a two-zinc ion center.

The ASM possesses conformational flexibility due to the saposin domain. This is very important for understanding the molecular mechanism of ASM function and how its domains play out together. The saposin domain can adopt one of two conformations: a globular closed form that interacts with the additional C-terminal subdomain, or an open V-shaped fold that establishes an extended interface with the catalytic domain, which is essential for the substrate hydrolysis. The ASM in solution presents an equilibrium between this two conformations of the saposin domain, the closed one in the absence of membranes that would turn the enzyme inactive, and the open one in the presence of negatively charged membranes (anionic membranes), consequently activating the sphingomyelin hydrolysis [39,40].

*SMPD1* mutations are distributed throughout the gene and the different locations in which the mutation takes place leads to different levels ASM enzymatic function impairment [39]. Most of these mutations are likely to destabilize the protein’s fold; however, there are also surface mutations that could affect the interaction of ASM with membranes and other proteins [40]. Many mutations affect hydrophobic residues in the β sheets regions and in the α-helices, which likely leads to the destabilization of the protein [39]. Regarding specific mutations, H319Y, H425R and D278A affect the active site of the enzyme, which leads to a complete loss of ASM activity [39]. Mutations C385R and C431R also affect the active site, however C385R presents a more severe effect than C431R [39]. Mutations in the saposin domain do not lead to total loss of ASM function, expect for the P184L mutation [39]. Mutations present in the tight association between the saposin domain and catalytic domain are mostly severe [39].

## 6. Acid Sphingomyelinase Role in the Immune System

Immune cell activation, following detection of pathogens or damage cells, is mediated by receptor binding and signaling. For receptor activation, ceramide-enriched membrane platforms are vital. ASM, by metabolizing sphingomyelin into ceramide at the cellular membrane, is critical for the development of ceramide-enriched membrane platforms and, therefore, cell-surface-receptor activation. In ceramide-enriched membrane platforms, receptors are clustered, allowing for complete receptor activation and potent signaling transduction [41].

Under physiological conditions, ASM is located in the lysosomes; however, following stress (including contact with pathogens and cytokines), the enzyme is secreted, hydrolyzing sphingomyelin present in the outer leaflet of membranes to ceramide, causing a re-structuration of the normal membrane rafts into larger structures [33,42]. It is within these structures that downstream signaling, through ceramide activity, occurs.

Along with the role of ASM in the promotion of ceramide-enriched platforms that occurs in a variety of immune cell types, ASM specifically controls the activity of invariant Natural Killer T (iNKT) cells [43]. ASM controls the activity of iNKT cells by degrading sphingomyelin that was recently shown to inhibit iNKT cell activation.

With this in mind, in this section, we here describe the current knowledge on the relevance of ASM activity in different cells of the immune system. The main effects of ASM on these cells are summarized on Table 1. Regarding other effects of ASM, besides its role on the immune system, more details can be found in recent review papers, namely Henry et al. 2013, Park et al. 2020 and Perrotta et al. 2015 [44,45,46].

### 6.1. Macrophages

ASM influences macrophage function in various ways, including promoting inflammatory response with cytokine production, promoting fusion of late phagosomes with lysosomes and regulating apoptosis. ASMD is particularly related with macrophage dysfunction, since these cells are one of the main targets in this disorder [47,48].

Macrophages from ASM knockout (KO) mice present an impaired uptake of the ASM enzyme via M-6-P receptors [47]. ASM leads to induction / amplification of inflammatory signals and cytokine production, in response to bacterial components [49,50,51] as well as oxidized LDL [52] and saturated fatty acid such as palmitic acid [50]. One prime example of this is the ASM role in the upregulation of inflammatory cytokine, IL-6 in response to LPS and palmitic acid [50]. Macrophage cell culture experiments with LPS and palmitic acid have demonstrated an increase in ASM activity with more production of ceramide, which contributes to the enhancement of the NF-kB dependent IL-6 expression [50]. Inhibition of ASM activity suppress inflammatory cytokine production from macrophages and protects animals against inflammatory diseases, such as experimental colitis and sepsis [49,52]. These results were corroborated by the treatment of macrophages with SMA-7, that is a ASM inhibitor, that caused a significantly decreased production of ceramide and release of inflammatory factors [53].

ASM is also essential for proper fusion of late phagosomes with lysosomes which is crucial for efficient transfer of lysosomal antibacterial into phagosomes [54]. This explains the increased susceptibility of ASM KO mice to intracellular pathogen infection, such as *Listeria monocytogenes* [55] and *Leishmania donovani* [56]. In particular, for *L. monocytogenes* infection of ASM KO mice, it was clearly shown that the increased susceptibility to infection was not associated with inadequate cytokine release, numerical alterations in the blood count or pathogen uptake, as these remained generally identical or close to the wild type results. The authors described that this macrophage failure to kill *L. monocytogenes* is due to the role of ASM in vesicle traffic and fusion [54].

Ceramide is a well-known pro-apoptotic agent in various cell types, including macrophages [57]. ASM, by producing ceramide, has been implicated in promoting macrophage apoptosis [57]. This was demonstrated in oxidized low-density lipoproteins (LDL)-induced macrophage apoptosis [58,59] and in *Pseudomonas aeruginosa*–induced macrophage apoptosis [60]. Human macrophage apoptosis, induced by oxidized LDL, was associated with increased ASM expression and ceramide concentration, whereas ASM inhibition leads to diminished oxidized LDL induced macrophage apoptosis [58]. ASM/ceramide facilitates oxidized LDL-induced macrophage apoptosis via endoplasmic reticulum stress pathway [59]. As many pathogens, *Pseudomonas aeruginosa* induces macrophage apoptosis. Alveolar macrophage apoptosis induced by *P. aeruginosa* is regulated by redox signaling, and ASM is critically involved in this process via production of ceramide-enriched membrane platforms [60]. Indeed, with infection, macrophages increase ASM activity, leading to ceramide production and formation of ceramide-enriched platforms, which are required for *P. aeruginosa*, induced activation of NADPH oxidase and production of reactive oxygen species. These ROSs further potentiate ASM activation and the creation of ceramide-enriched platforms, which culminate in the induction of apoptosis via a JNK-dependent pathway [60].

The proteins secreted by activated macrophages in plasma of patients with ASMD, such as chitotriosidase, can also be of great relevance as clinical biomarkers [61]. Chitotriosidase presents elevated levels in ASMD but can have a rapid decrease upon treatment [62].

### 6.2. NK Cells

NK cells play a major role in innate immunity. ASM influences NK cell functionality via CD161 [63], a major phenotypic marker of these cells, by interacting with intracellular region of CD161. The veracity of this interaction was further solidified by experiments where the crosslinking of CD161 with anti-CD161 antibodies in primary human NK cell lines was shown to lead to the activation and recruitment of ASM towards the mentioned marker [63]. The consequent generation of ceramide, in turn, acts as a second messenger that activates various NK cell signaling pathways, such as PKB/Akt and Rsk1/MAPKAP-kinase 1α [63]. Importantly, treatment of cells with an ASM inhibitor blocks PKB and Rsk1 activation. ASM was shown to also be critical for the maintenance of the costimulatory functions of CD161 for cell proliferation. ASM inhibition with imipramine leads to loss of co-stimulation activity, similar to the findings of similar experiments that looked into the co-stimulation of INF-γ by CD161 [63].

**Table 1:** ASM effects on immune-system cells.

Cell Type	Effect of ASM	References
Macrophages	Induces/amplifies inflammatory signals with cytokine production. Promotes proper fusion of late phagosomes with lysosomes. Promotes macrophage apoptosis.	[34,35,36,37,38], [41]
NK Cells	Influences these cells function via CD161. Leads to activation of NK cell signaling pathways. Is involved in NK cell apoptosis.	[45,46]
B Cells	Mediates CD40 clustering and in this way mediates B cell activation. Is involved with plasma membrane damage repair. Important for autophagic function.	[48,51], [52,53]
CD4^+^ T Cells	Involved in TCR mediated activation. Involved with polarization into subtypes Th1, Th2 and Th17. Acts a negative regulator of Tregs.	[54,58,59,60], [62]
CD8^+^ T Cells	Involved in the cellular membrane’s biophysical properties inducing the extrusion of lytic granules from the cells by promoting secretory granules contraction.	[64]
iNKT Cells	Involved in iNKT cell development and activation. ASM substrate sphingomyelin impedes CD1d access to antigenic lipids, thus reducing iNKT activation.	[33]

Parallel to its role in macrophage apoptosis, ASM seems to be involved also in NK programmed cell death. ASM-dependent ceramide elevation is induced by IL-2 deprivation in an human NK cell line, leading to apoptosis [64]. The mechanism behind this event is ceramide-mediated X-linked inhibitor of apoptosis protein (XIAP) degradation by activating cytosolic cathepsin B (CTSB) and caspase dependent apoptosis [65]. Conversely, IL-2 action leads to NK cell survival by inhibiting of ASM and enhancing glucosyl ceramide synthase, leading to a direct increase in the levels of sphingomyelin and ceramide [64].

### 6.3. B Cells

B cells are a part of one of the big branches of adaptive immunity, being responsible for antibody-mediated immunity. Moreover, they can also act as antigen-presenting cells, capable of processing and presenting antigens to other cells of the immune system. ASM has been shown to have an effect on antigen presenting cells, as it has been demonstrated to be crucial for the clustering of CD40 [66], a costimulatory protein present in these cells that is essential for their activation. In addition, CD40 has been shown to be central in the stimulation of B cells, as B cells lacking signals via CD40 are unable to switch from IgM to IgG synthesis [67,68].

ASM translocation to the extracellular surface of the cellular membrane happens upon CD40 ligation, which then allows for the release of ceramide that will mediate CD40 clustering [66]. The evidence for this mechanism is further supported by the fact that both neutralization of surface ceramide and ASM deficiency lead to the impairment of CD40 clustering and, consequently, CD40 cell signaling in B cells [66].

ASM was also shown to be involved with B-cell plasma membrane damage repair [69]. This process, which happens in a Ca^2+^ dependent manner, inhibits B-cell activation through B-cell receptor signaling and vice versa. This is believed to happen due to the fact that both processes require lipid rafts and seem to not be able to take place concomitantly [69]. The resealing process of plasma repair was shown to be reduced both by an ASM reduction and ASM inhibition, as well as restored and even enhanced by extracellular sphingomyelinase exposure [69].

B cells have also been shown to be defective in various processes, such as autophagy, mitochondrial clearance and lipophagy in a cellular model of ASMD B, consisting of a B-lymphocyte cell line derived from an ASMD B patient [70]. The authors found that ASMD B cells present an accumulation of partially degraded mitochondrial fragments, as well as an atypical pattern of autophagy, with a big number of autophagic vacuoles. The accumulation of these vacuoles is seen as a general manifestation of autophagic stress [70]. These B cells displayed autophagic dysfunction both in the initial and final phases of the process, as well as peroxidized lipid droplet accumulation and increased levels of ROS. Canonico and colleagues also investigated the possible actions of rapamycin on these phenotypes in B cells, as this drug has been previously shown to have potential as therapeutic agent with the capability for autophagy induction [71]. In fact, rapamycin was shown to reduce both mitochondrial and intracellular ROS, as well as regulate mitophagic vesicle formation and lipid droplet accumulation, leading to the maintenance of cell viability [70]. Because of these findings, the authors suggest that rapamycin could be used in pharmacological schemes for LSDs, such as ASMD B, because of its ability to regulate autophagic imbalances, even if only partly.

### 6.4. CD4^+^ T Cells

CD4^+^ T cells are a type of T lymphocytes that play a major role in mediating immune response through the secretion of specific cytokines. CD4^+^ T cells can polarize into distinct effector subtypes. Some of these subtypes that have been demonstrated to be affected by ASM function are T-helper 1 (Th1) cells, T-helper 2 (Th2) cells, T-helper 17 (Th17) cells and T-regulatory cells (Treg) [41]. The CD4^+^ T cells present multiple functions essential for the regulation of the immune system. These include activation of a range of cells (B-lymphocytes, cytotoxic T cells and nonimmune cells) and suppression of immune reaction, in which Treg cells play a major role. These last cells are one of the most talked about subtypes in regard to its relationship with ASM. Tregs depend on the signaling of CD28 for their survival and function. CD28 has been previously demonstrated to activate the ASM system [72].

Regarding the effect of ASM on CD4^+^ T cells, the mechanisms of these interactions are still poorly understood. However, in recent years, various articles have been trying to uncover these interactions and potential outcomes [41]. Using the ASM KO mice as a model, investigators are trying to elucidate the role of ASM in regard to several processes in vivo. In vitro, pharmacological inhibition of ASM in T cells is the most commonly used model.

ASM was found to be involved in CD4^+^ T-cell receptor (TCR)-mediated activation and, in this way, has an impact on the immune response [73]. ASM interacts with intracellular domains of CD3 and CD28 and mediates its signaling, as pharmacological inhibition or knockdown ASM blocks CD3/CD28 signaling cascades and consequently inhibits the activation and proliferation of human CD4^+^ T cells [73]. This holds true for both naïve and memory CD4^+^ T cells. Furthermore, this inhibition of ASM activity was demonstrated to inhibit the polarization of CD4^+^ T cells into Th1, Th2 or Th17 in vitro, measured by the lower level of IFN-γ, IL-4 and IL-17 cytokine production, respectively [73]. A similar conclusion on the role of ASM in promoting T-cell activation was drawn from a mouse model overexpressing ASM on T cells [74]. T-cell-specific ASM overexpressing mice revealed elevated TCR signaling activity and increased proliferation upon stimulation in vitro [74]. In vitro, this enforced T cell specific ASM expression also led to a promotion of differentiation of naïve T cell into IFN-γ-producing Th1 cells [74].

Recently, the role of ASM in the pathology of asthma was investigated in ASM-deficient mice [75]. At baseline, the bronchoalveolar lavage fluid is enriched in CD4^+^ T cells that produce lower levels of Th2 cytokine IL-4. Consequently, ASM KO mice were protected from airway hyper-responsiveness, in an ovalbumin asthma model, due to decreased Th2 response [75]. This could be explained by impaired function of secretory pathways in ASM-deficient mice. In contrast, in an animal model of pathogen-driven colitis, ASM deficiency contributes to the increase in pathology [76]. The absence of ASM activity in mice infected with enteric pathogen *Citrobacter rodentium* leads to higher infection and colitis pathology associated with increased proportions of Th1 and Th17 cells in the intestinal lamina propria [76].

ASM activity is higher in Tregs than in conventional CD4^+^ T cells of wild-type mice [72]. ASM KO mice have a significant higher frequency of splenic Tregs [77] and Tregs with higher suppressive activity in vitro compared to control mice [72]. In addition, in vitro induction of Tregs with TGF-β and IL-2 is increased in ASM-deficient T cells. ASM seems to be a negative regulator of both natural and induced Tregs. To corroborate this result, another study also demonstrated this effect of Tregs in vivo and an increase of their suppressive activity in vitro [72]. The same investigation found the same results in wild-type mice treated with an ASM inhibitor [72]. However, they went further into exploring the effect of ASM in CD4^+^ cells and observed a reduced absolute cell number of conventional CD4^+^ T cells after inhibitor treatment in vivo [72]. Moreover, in a study regarding the role of ASM in the regulation of tumor immunogenic microenvironment, using in vivo melanoma models, in the absence of ASM, high levels of Tregs were, once again, observed [78]. Concomitantly, decreased frequencies of Tregs in the spleen were observed in mice with T-cell-specific ASM overexpression [74]. Regarding this, there is already a study performed in humans that supports these findings [79]. In this study the pharmacological inhibition of ASM increased the frequency of Tregs among human CD4^+^ T cells [79].

The absence of ASM in mice has also been correlated with the abolishing of the partial protection that effector memory CD4^+^ T cells present against glucocorticoid-induced cell death [80]. This increased susceptibility to glucocorticoid-induced cell death in ASM-deficient mice could be due to a reduced secretion of IL-2 by CD4^+^ T cells, especially since the addition of IL-2 restored this protective feature [80]. In fact, the stimulation of splenocytes in wild-type mice led to the increase of IL-2 expression. However, in ASM KO mice, the levels of secretion of IL-2 were reduced; however the intracellular levels of this cytokine were elevated [81].

### 6.5. CD8^+^ T Cells

Naïve CD8^+^ T cells differentiate into cytotoxic T lymphocytes upon antigen recognition. Cytotoxic T lymphocytes (CTL) express lytic granules. The release of these granules is stimulated by agonistic ligation of TCR to the corresponding antigen at the surface of antigen presenting cells. Lytic granules contain granzyme A and granzyme B and perforin, which generates pores in the target cell’s membrane through which granzymes can enter the cytoplasm and induce apoptosis by activating caspases. 

ASM is involved in the function of the CTL by inducing granule secretion [53,82]. The release of the cytotoxic granules from CTL is defective in ASM-deficient mice [82]. This defect delays the clearance of the lymphocytic choriomeningitis virus [82] from these mice. The contents of granzyme A, granzyme B, and perforin mRNA, as well as protein and enzymatic activity, remain the same in the ASM-deficient mice and the wild-type mice [82]. The antigen-specific activation and proliferation of CD8^+^ T cells in both mice were also normal. However, in the ASM-deficient mice, there is a defect in the contraction of secretory granules, presenting accumulation of larger clusters than the wild-type mice of granzyme-positive granules directly adjacent to the immunologic synapse [82]. When ASM is not present, the size of vesicles is only reduced by 44% after fusion with the plasma membrane, which causes a lower efficiency of the release of cytotoxic effector molecules [82]. These data were confirmed by pharmacological inhibition of ASM activity in CTL [82]. These changes were mediated by the absence of ASM generating ceramide, which alters the cellular membrane’s biophysical properties, namely surface tension of membrane leaflets, impairing extrusion of lytic granules from the cells [53].

These results support a role of ASM in CTL granules release and points to a possible use of ASM inhibitors in clinical conditions of immunopathology due to overshooting CTL activity. Indeed, the role of ASM inhibition in patients with cystic fibrosis suffering from bacterial infection of the lung has been investigated in clinical trials [83].

### 6.6. iNKT Cells

Invariant Natural Killer T (iNKT) cells are lipid reactive T cells bearing a limited TCR repertoire being restricted to the MHC class-I-like CD1d molecule [84,85]. Both self and foreign lipid ligands presented by CD1d control iNKT cell function. A key characteristic of these cells is the rapid release of cytokines upon activation, which prompts them to control innate and adaptive immune responses by other immune cell types downstream. iNKT cells are of great importance in controlling tumor growth and infection [84,85].

Moreover, iNKT cell activation is thought to be influenced by the balance of CD1d-associated antigenic and non-antigenic lipids, but very little was known about the functional relevance of non-antigenic lipids that potentially impede CD1d-restricted NKT cell activation [85,86].

**Figure 2:**
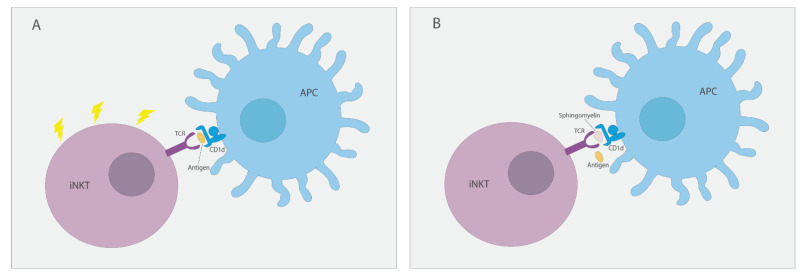
Sphingomyelin regulates CD1d access to potentially agonistic lipids. (**A**) In the absence of sphingomyelin, lipid antigens are free to access CD1d present in antigen-presenting cells (APCs), binding to iNKT cell TCR and leading to cell activation. (**B**) In cases of ASM deficiency, excess sphingomyelin binds to CD1d on APCs, preventing the binding of lipid antigens, and, as a consequence, leads to impediment of iNKT cell activation.

Recently, ASM was shown to be involved in iNKT cell development and activation in an elegant study showing that the ASM substrate sphingomyelin regulates CD1d access to potentially agonistic lipids [43]. Both ASM KO mice and ASM-deficient patients’ cells were analyzed in this study. In mice, the absence of ASM leads to diminished CD1d-restricted endogenous and exogenous lipid-antigen presentation to iNKT cells. This, in turn, debilitates iNKT cell development in the thymus that leads to reduced iNKT cell levels in the thymus and systemic. A schematic representation of proposed mechanism can be observed in Figure 2. Importantly, ASM deficiency in ASMD patients also leads to decreased lipid-antigen presentation by CD1d and lower peripheral blood iNKT cell numbers. These alterations were shown to be reversible by ASM replacement therapy in young ASM KO mice [43]. ASM therapeutic effect in ASM KO mice provides confidence on the effect of ASM therapy in ASMD patients’ pulmonary infections. These infections are common comorbidities of this disease, and iNKT cells have a critical role in antimicrobial immunity against common respiratory pathogens, such *Pneumococcus* and *Pseudomonas* [87,88].

## 7. Susceptibility to Lung Disease

Lung disease is a common clinical feature in patients with ASMD, being one of the most important causes of morbidity and mortality [5,7,89,90,91,92,93]. ASM KO mice recapitulate the lung disease present in ASMD patients [94]. The pathophysiology of the pulmonary disease is likely related to the accumulation of sphingomyelin in alveolar macrophages. There is an increased cellularity in ASM KO mice lungs, mainly macrophages, but also neutrophils in older mice. This increased cellularity is accompanied by elevated cytokine expression and generalized inflammation [94].

Interestingly, even a 50% reduction of ASM activity in bronchial epithelial cells (using inducible shRNA) leads to increased inflammation both in unstimulated and infected conditions [95]. The decreasing ASM activity leads to an increased neutrophil recruitment via elevated levels of cytokine expression, both at baseline and in response to bacterial stimulation, supporting the hypothesis of a chronic inflammatory state impairing host defense mechanisms [95]. In line with these results, studies of *Pseudomonas aeruginosa* ASM KO mice infection reveal massive inflammation associated with IL-1 release and septic death of mice. This is due to the physiological role of ASM in regulating immune response to *P. aeruginosa*. Upon *P. aeruginosa* infection, there is an activation of ASM with release of ceramide, leading to the production of larger signaling platforms that are required for bacteria internalization, infected cell apoptosis and regulation of cytokine release from infected cells [96].

The exaggerated inflammatory response in ASM KO mice occurs not only in response to *P. aeruginosa* but also in response to polymicrobial sepsis [97]. ASM KO mice have a more pronounced cytokine storm and sepsis in response to peritoneal contamination with stool suspension [97].

Along with mouse studies, the clinical history of ASMD patients also points to a highest susceptibility to lung disease, with some patients presenting respiratory failure [5,89,91,92]. The progression of respiratory disease is slow but inexorable, due to the accumulation of lipid laden macrophages in the respiratory system [89,91]. Along with lipid laden macrophages, bronchoalveolar lavage results demonstrate the presence of local inflammation, which may also contribute to the respiratory manifestations observed in these patients [91]. Importantly, ASMD patients present frequent respiratory infections, including pneumonia, and pneumonia is a leading cause of death [5,7,90].

## 8. Monitoring and Treatment

On the market, there are no approved specific therapies for ASMD. Therefore, currently, management of this disease is directed to reducing the impact of the multisystemic disease symptoms. The monitoring assessments and treatments associated to each clinical manifestation were recently described in “Recommendations for clinical monitoring of patients with acid sphingomyelinase deficiency (ASMD)” [98].

At present, enzyme replacement therapy (ERT) using human recombinant acid sphingomyelinase—olipudase alfa—is in clinical development for the treatment of non-neurological manifestations of ASMD. Two clinical trials are currently being conducted and are in phase 2 (NCT02004704) and 2/3 (NCT02004691). In addition, three studies have been completed, one in phase 1/2 (NCT02292654) and the other two in phase 1 (NCT00410566; NCT01722526).

A phase 1 single-ascending-dose study of olipudase alfa, aiming to address the safety of ASM ERT in adult patients, identifies 0.6 mg/kg as the maximum tolerated first dose (NCT00410566; [99]). Eleven patients participated in this study divided into five groups that were infused with different doses of olipudase alfa (0.03, 0.1, 0.3, 0.6 or 1.0 mg/kg). No serious adverse drug reactions occurred during the study. Acute-phase reaction-type adverse drug reactions arose 12–24 h following doses higher or equal to 0.3 mg/kg. Three patients developed hyperbilirubinemia. The patient receiving 1 mg/kg experienced severe hyperbilirubinemia; he was subsequently diagnosed with Gilbert syndrome [99]. The nature and timing of the first dose adverse drug reactions are consistent with an acute inflammatory response by the innate immune system. Following olipudase alfa treatment, a possible initiator of inflammation is ceramide, a well-known signaling molecule involved in inflammation and produced by the ASM activity. Indeed, plasma ceramide showed an elevation by 6 h post-infusion, preceding the onset of symptoms and elevation of bilirubin [99]. Therefore, the first-dose adverse drug reactions were likely induced by elevated ceramide or its metabolites, generated by the catabolism of accumulated sphingomyelin. It was hypothesized that a patient dose escalation would slow down the production of ceramide and may reduce adverse drug effects of olipudase alfa. Consequently, a within-patient dose-escalation study was designed and conducted (NCT01722526; [100]). In this study, to ensure the safety of the patients, an initial dose of 0.1 mg/kg was used. After two weeks, the dose was changed to 0.3 mg/kg, which had to be repeated two weeks later for verification of tolerability. With the same time interval, the doses escalated to 0.6, 1 and 2 mg/kg, ultimately achieving the target dose of 3 mg/kg. This dose was maintained until the end of the study (26 weeks) [100]. From the five patients that participated in this trial, only two manifested symptoms similar to an acute phase reaction, but only one had to undergo a dose reduction. However, both achieved the target dose at the end of the study. None of the patients manifested severe adverse events, with the most common events being classified as mild (97%). The common treatment-emergent adverse events were headache, arthralgia, nausea, abdominal and back pain, pain in the extremities, abdominal discomfort and pyrexia. However, there were six moderate adverse events in two patients related with infusion-associated reactions [100]. Good clinical results were also observed and included decreased liver and spleen volume (13.4% and 25.3%, respectively) and improvements in infiltrative lung disease, platelet counts and quality of life [100]. Liver sphingomyelin storage had a reduction between 84% and 92%, and the lipid-plasma profile also presented significant improvements. Total cholesterol, LDL-C, VLDL-C and triglycerides decreased achieving normal ranges. On the other hand, HDL-C increased in 13% to 84% [100,101]. In a similar way, a study in phase 1/2 was conducted in pediatric patients in order to evaluate the safety and tolerability of olipudase alfa with ascending doses (NCT02292654; [61]. Twenty patients were enrolled in this study, with ages between 1.5 and 17.5 years. The first infusion started at 0.03 mg/kg and followed by 0.1 mg/kg two weeks after. Latter, escalating doses were given: 0.3, 0.6, 1 and 2 mg/kg, reaching, at last, the maintenance dose of 3 mg/kg by week 64. All patients reported at least one mild adverse event, such as pyrexia, cough, vomiting, nasopharyngitis, diarrhea, headache, nausea, rhinitis, oropharyngeal pain, ear pain and rhinorrhea [61]. However, five patients reported a serious adverse events related to the treatment, one of which led to a temporarily discontinuation. This case occurred in a 17-month-old infant due to an anaphylactic reaction. After desensitization, dose escalation was resumed and the maintenance dose was reached by the end of the study. Overall, the treatment was well-tolerated, and the associated with clinically meaningful improvements has already demonstrated in adult patients.

As stated before, two clinical trials, one phase 2 and one phase 2/3 (NCT02004704; NCT02004691), are still ongoing with olipudase alfa treatment once every two weeks. The outcomes from the past 30 months show that olipudase alfa is well tolerated and inductive of extensive improvements in relevant disease clinical measures [102], including significant reductions on liver (31%) and spleen (39%) volumes and an increase of 35% in lung diffusion capacity. Lipid profiles improved in all patients: triglycerides decreased by 43%, total cholesterol by 13%, LDL-C decreased by 23% and HDL-C increased by 138%. In terms of safety, there were no serious or severe events during treatment. Vital signs, hematology and cardiac safety parameters were maintained within normal ranges throughout the study [102]. Subsequent analyses at 42 months of treatment confirm the long-term efficacy of olipudase alfa in the improvement of the lipid profile of ASMD patients [103]. Indeed, a progressive clearance of sphingomyelin storage in the liver, improvement in liver enzymes and progressive reduction of total cholesterol, LDL-C, VLDL-C and triglycerides were observed. Conversely, HDL-C increased up to 200% [103].

Recently, the outcomes of phase 2/3 clinical trial with olipudase alfa were published in a press release from Sanofi (NCT02004691; NCT02292654). Treatment with olipudase alfa demonstrated 22% of lung function improvement (using diffusing capacity of carbon monoxide) and 39.5% reduction on spleen volume relative to baseline. The difference between olipudase alfa and the placebo was statistically significant. In terms of adverse events, there were 242 (three severe and five serious) with olipudase alfa and 267 (13 severe and 11 serious) with the placebo (NCT02004691; NCT02292654).

## 9. Conclusions

The development of recombinant acid sphingomyelinase for the treatment of acid sphingomyelinase deficiency awakened the interest on this rare lysosomal disease. The clinical, biochemical, cellular and molecular presentation of this disease is underscored in the present review. In particular, the composition and location of the characteristic foam cell infiltration are described, along with the hepatosplenomegaly, lung dysfunction and central nervous system clinical presentation. Acid sphingomyelinase deficiency is a pan-ethic disease associated with more than 200 *SMPD1* gene variants. Even though *SMPD1* gene mutations are highly heterogeneous, some genotype/phenotype association have been described.

Acid sphingomyelinase has a critical role in different cells of the immune system, mainly due to its role in the promotion of ceramide-enriched platforms. However, acid sphingomyelinase has a specific role in controlling the activity of Natural Killer T (NKT) cells by controlling the availability of sphingomyelin that was recently described as a lipid able to restrain NKT cell activation. With the availability of enzyme replacement therapy, for this disease, better clarification of the role of acid sphingomyelinase in the immune response is expected. Therefore, exciting times are approaching both clinically and scientifically for this lysosomal storage disorder.

## Data Availability

Not applicable.
